# Perindopril Improves Cardiac Function by Enhancing the Expression of SIRT3 and PGC-1α in a Rat Model of Isoproterenol-Induced Cardiomyopathy

**DOI:** 10.3389/fphar.2020.00094

**Published:** 2020-02-21

**Authors:** Zhenyu Zhu, Huihui Li, Wanli Chen, Yameng Cui, Anan Huang, Xin Qi

**Affiliations:** ^1^ School of Graduate Studies, Tianjin University of Traditional Chinese Medicine, Tianjin, China; ^2^ Department of Cardiology, Tianjin Union Medical Center, Tianjin, China; ^3^ School of Medicine, Nankai University, Tianjin, China

**Keywords:** perindopril, heart failure, PGC-1α, mitochondrial biosynthesis, oxidative stress, apoptosis

## Abstract

Mitochondrial biosynthesis regulated by the PGC-1α-NRF1-TFAM pathway is considered a novel potential therapeutic target to treat heart failure (HF). Perindopril (PER) is an angiotensin-converting enzyme inhibitor that has proven efficacy in the prevention of HF; however, its mechanism is not well established. In this study, to investigate the mechanisms of PER in cardiac protection, a rat model of cardiomyopathy was established by continuous isoproterenol (ISO) stimulation. Changes in the body weight, heart weight index, echocardiography, histological staining, mitochondrial microstructure, and biochemical indicators were examined. Our results demonstrate that PER reduced myocardial remodeling, inhibited deterioration of cardiac function, and delayed HF onset in rats with ISO-induced cardiomyopathy. PER markedly reduced reactive oxygen species (ROS) production, increased the levels of antioxidant enzymes, inhibited mitochondrial structural destruction and increases the number of mitochondria, improved the function of the mitochondrial respiratory chain, and promoted ATP production in myocardial tissues. In addition, PER inhibited cytochrome C release in mitochondria and caspase-3 activation in the cytosol, thereby reducing the apoptosis of myocardial cells. Notably, PER remarkably up-regulated the mRNA and protein expression levels of Sirtuin 3 (SIRT3), peroxisome proliferator-activated receptor gamma coactivator 1-alpha (PGC-1α), nuclear respiratory factor 1 (NRF1), and mitochondrial transcription factor A (TFAM) in myocardial cells. Collectively, our results suggest that PER induces mitochondrial biosynthesis-mediated enhancement of SIRT3 and PGC-1α expression, thereby improving the cardiac function in rats with ISO-induced cardiomyopathy.

## Introduction

Congestive heart failure (HF) is a common progressive disease, and consequently, expanding efforts are being made to develop novel treatments to intervene with its progression and prevent its occurrence (Povsic, 2017). During HF development, mitochondrial dysfunction in cardiomyocytes is evidenced by decreased cytochrome oxidase activity; and insufficient oxidation of nicotinamide adenine dinucleotide (NADH) can induce the production of reactive oxygen species (ROS) (Bond et al., 2019). Angiotensin II (AngII) induces accumulation of intracellular ROS by activating nicotinamide adenine dinucleotide phosphate (NADPH) oxidase, eventually leading to myocardial remodeling and HF (Idris-Khodja et al., 2013). Excessive ROS production is recognized to accelerate HF progression (Wrigley et al., 2011). Thus, a current focus in the treatment of hypertension is identifying treatment modalities that improve mitochondrial health (Eirin et al., 2018).

One approach toward improving mitochondrial health involves the activation of mitochondrial biosynthesis, which is of vital importance in maintaining mitochondrial structure and function. Mitochondrial biosynthesis is regulated by the peroxisome proliferator-activated receptor gamma coactivator 1-alpha (PGC-1α) (Cheng et al., 2016). Reduced PGC-1α expression is observed in various HF models and is accompanied by oxidative stress (OS) and mitochondrial dysfunction (Piquereau et al., 2017; Waldman et al., 2018). According to a recent study (Parodi-Rullán et al., 2018), sirtuin 3 (SIRT3) mediates the deacetylation of forkhead box O3 and induces its nuclear translocation, which up-regulates PGC-1α expression. Evidence (Singh et al., 2018; Waldman et al., 2018) suggests that regulation of diabetic cardiomyopathy in a mouse model by either caloric restriction or administration of epoxyeicosatrienoic acid-agonist involves the induction of PGC-1α. Additionally, activation of PGC-1α has been associated with the protective effects of qiliqiangxin capsule in a pressure overload heart failure model (Zhang et al., 2013) and adiponectin in an iron-overload cardiomyopathy model (Lin et al., 2013). Mitochondrial biosynthesis regulated by the PGC-1α-NRF1-TFAM pathway has also been suggested as the primary mechanism of Carvedilol, a nonselective β-adrenoceptor antagonist that has proven clinical efficacy (Yao et al., 2016). Therefore, ample evidence supports the therapeutic potential of drugs that target the PGC-1α pathway.

Angiotensin-converting enzyme (ACE) inhibitors are an important class of therapeutic agents for HF that relax blood vessels and lower blood pressure, thus facilitating blood flow (Nabeebaccus et al., 2016). Evidence suggests that the ACE inhibitor lisinopril modulates age-related mitochondrial metabolic parameters in Drosophila melanogaster (Ederer et al., 2018) and exercise-induced mitochondrial gene transcript expression in human volunteers (van Ginkel et al., 2016). Furthermore, zofenoprilat has been shown to regulate angiotensin I receptor expression through SIRT1 downregulation (Marampon et al., 2013). Enalapril increases mitochondrial production of NO and regulates ROS production in the rat heart (Costa et al., 2002), and recent evidence suggests that late life enalapril administration is associated with increased TRAM activity (Picca et al., 2018). Therefore, it is likely that the therapeutic efficacy of ACE inhibitors stems from their mitochondrial protective effects, and that mitochondrial biosynthesis may be a pivotal component.

Perindopril (PER), an extensively used long-acting ACE inhibitor, has been demonstrated in a number of large-scale clinical trials to be effective in myocardial remodeling, improvement of cardiac function, and reduction of long-term HF-related mortality (Bertrand et al., 2009). However, to the best of our knowledge, it remains unclear whether PER might facilitate mitochondrial biogenesis to improve cardiac function and delay HF progression. In this study, we established a rat model of isoproterenol (ISO)-induced cardiomyopathy to investigate the potential mechanism of PER in improving cardiac protection through the SIRT3/PGC-1α pathway.

## Materials and Methods

### Animal Model and Treatment Protocols

Our animal experimental protocol was approved by the Animal Care and Use Committee of Tianjin Union Medical Center. A total of 36 male adult Sprague-Dawley (SD) rats (weight, 200–250 g) were used in this study. The rats were provided by the Vital River Laboratory Animal Technology Co., Ltd. (Beijing, China). All animals were adapted to the new environment for 1 week prior to experiments and were provided free access to foods and water under standard laboratory conditions. The animals were randomized into two groups: the ISO-induced cardiomyopathy group (n = 26) and the control group (n = 10). The ISO-induced cardiomyopathy group was given subcutaneous injection of ISO (Purity 98%, Beijing Solarbio Science & Technology Co., Ltd, China) dissolved in normal saline at 10 mg/kg/d once daily for 2 consecutive weeks. The control group was given injection of normal saline in parallel. Afterwards, 20 surviving animals from the ISO-induced cardiomyopathy group were further divided into 2 groups: the ISO + PER (n = 10) group and the ISO (n = 10) group. Animals in the ISO + PER group were given PER (SFDA Approval Number H20103382, 8 mg/tablet, Seriver (Tianjin) Pharmaceutical Co., Ltd, Tianjin, China) gavage at 2.0 mg/kg daily, while those in the ISO and control groups were given normal saline gavage at an identical volume once a day for six consecutive weeks.

At the end of week 8, the animals were euthanized, and blood samples were collected into tubes. At the same time, fresh cardiac tissues were collected and rinsed with cold saline. A portion of the tissues was utilized for mitochondrial protein extraction, while another part was used for myoblast suspension preparation to determine ROS and mitochondrial membrane potential (MMP) levels. The remaining tissues were preserved at −80°C for subsequent use or fixed using 4% paraformaldehyde, followed by paraffin embedding for pathological and transferase dUTP nick end labeling (TUNEL) staining.

### Echocardiography

At weeks 2 and 8, the animals were injected with 3% sodium pentobarbital solution at 10.0 mg/kg for anesthesia before echocardiographic measurements. Measurements of the left ventricular end diastolic diameter (LVEDD), left ventricular end systolic diameter (LVESD), left ventricular end, left ventricular ejection fraction (LVEF%), and left ventricular fractional shortening (LVFS%) were determined based on long-axis M-mode echocardiography using the Vevo2100 imaging system (VisualSonics, Toronto, ON, Canada) (n = 6).

### Heart Weight Index (HWI) Assessment

The excised cardiac tissues were washed with ice-cold PBS; subsequently, the adipose tissue, aorta and atria were separated, and the heart weight (mg) was measured. The HWI (mg/g) was defined as the ratio of heart weight to body weight (n = 10).

### Histopathological Analysis

Ventricular tissues were fixed in 4% paraformaldehyde, followed by paraffin embedding, sectioning (5 μm in thickness), and staining using Masson’s trichrome and hematoxylin-eosin (H&E) solution. Finally, the collagen content and morphological changes in the cardiac tissues were assessed by light microscopy (DS-Ri2, Nikon, Japan) (n = 5). Image J was used to analyze the images of pathological tissues and the Collagen Volume Fraction (CVF) of myocardial tissues was calculated based on the formula: CVF=Collagen area/Total observed area x100%.

### Measurement of Serum B-Type Natriuretic Peptide (BNP) and AngII Levels

After standing for 30 min, blood samples were centrifuged for 15 min at 3,000 g to separate the serum. Thereafter, the serum levels of B-type natriuretic peptide (BNP) and Ang II were determined by ELISA (Elabscience Biotechnology Co., Ltd, Wuhan, China) (n = 6). Configured standard working solution of 100 μl and test serum were added to each well of ELISA plate. Then, 50 μl of the prepared biotinylated antibody working solution to was immediately added to each well. The ELISA plate was covered with a membrane and incubated at 37°C for 45 min. The solution in the ELISA plate was discarded and the plate was washed three times. Enzyme conjugate working solution of 100 μl was added to each well, and the plate was placed in an incubator at 37°C for 30 min. The solution in the ELISA plate was discarded and it was washed 5 times. Then, 90 μl of substrate solution was added to each well and incubated at 37°C in the dark for 15 min. Finally, 50 μl of Stop Solution was added to each well. The optical density (OD) of each well was measured with a microplate reader (Epoch2, BioTek Instruments, Inc, America) at a wavelength of 450 nm. The calibration curves were generated with OD values as the Y axis and standard concentrations as the X axis. The concentration of BNP and Ang II were calculated by OD values on the basis of the calibration curves.

### Assessment of Oxidant/Antioxidant Activities

Cardiac tissues (10% w/v) were homogenized in cold saline, followed by 10 min centrifugation at 3,000 g to collect the supernatant. Subsequently, the contents of malondialdehyde (MDA), glutathione peroxidase (GSH-Px), and manganese superoxide dismutase (Mn-SOD) were measured in accordance with reagent protocols (Nanjing Jiancheng Bioengineering Institute, China) (n = 6).

### Transmission Electron Microscopy

From each group the cardiac tissues were minced into small pieces (≤1 mm^3^) and fixed in 2.5% glutaraldehyde in 0.1 mol/l sodium cacodylate buffer (pH 7.3) for 2 h. The specimens were rinsed in buffer, post-fixed in cacodylate-buffered 2% OsO^4^, stained en bloc in uranyl acetate, dehydrated gradient in ethanol, and embedded in epoxy resin. The samples were observed under a transmission electron microscope (JEM-1200, JEOL Ltd., Tokyo, Japan) (n = 3).

### Measurement of Mitochondrial Complexes I, II, III, and IV Activities

Myocardial mitochondria were extracted, and the mitochondrial respiratory chain complexes I, II, III, and IV activities were measured using corresponding Activity Assay kits (Beijing Solarbio Science & Technology Co., Ltd, China) according to the manufacturer protocols (n = 6).

### Measurement of ATP Contents

Cardiac tissues (10% w/v) were homogenized using boiling-hot double distilled water: The samples were then placed in a boiling water bath for 10 min, followed by 10 min centrifugation at 3,500 g to collect the supernatants. Thereafter, the ATP levels were measured according to the reagent protocol (Nanjing Jiancheng Bioengineering Institute, China) (n = 6).

### Flow Cytometry

Cardiac tissues were cut into 1 mm^3^ pieces, digested with trypsin in a constant temperature water bath (37°C) for 30 min, and filtered with a 300-mesh nylon filter. Afterwards, the filtrate was centrifuged at 600×g for 5 min at 4°C, the supernatant was discarded, and the sediment was re-suspended in cold PBS. These steps were repeated three times. Then, the myoblast suspension was loaded using DCFH-DA and JC-1 probes in accordance with the Active Oxygen Detection kit (Nanjing Jiancheng Bioengineering Institute, China) and the Mitochondrial Membrane Potential Assay kit (Beyotime Biotechnology, Shanghai, China). The myoblasts were washed with PBS three times after incubation at 37°C; finally, the DCF and JC-1 fluorescence intensities were measured by flow cytometry using the Guava EasyCyte flow cytometer (EMD Millipore Corporation, Hayward, Calif) (n = 3).

### TUNEL Assays

Apoptosis levels were determined by the TUNEL assay using the One Step TUNEL Apoptosis Assay kit (Dalian Meilun Biotechnology Co., LTD, China) according to the manufacturer protocol. Myocardial tissue sections were baked in an oven at 60°C for 30 min, deparaffinized with xylene (5 min, 3 times) and dehydrated with 100%, 95%, and 70% ethanol 3 times each. Next, the sections were incubated with protein kinase K for 30 min. After rinsing with Phosphate-Buffered Saline (PBS), TUNEL detection solution (including TdT and fluorescein-dUTP) was added for reaction at 37°C for 1 h. After washing with PBS 5 times, 1 μg/ml of DAPI the samples were incubated in the dark for 10 min at 37°C. Finally, tissues were washed with PBS and sealed with antifade solution (Solarbio, Beijing, China) and then observed with an Ortho fluorescence microscope (DS-Ri2, Nikon, Japan). Cells with green nuclei were deemed positive for apoptosis. Five representative fields were randomly selected at ×200 magnification. The apoptosis rate was calculated as the ratio of TUNEL positive cardiomyocytes nuclei to the total number of cardiomyocytes nuclei (n = 5).

### RT-PCR Assays

Total RNA was isolated from myocardial tissue using TRIzol reagent (Tiangen Biotech Co., Ltd, China), and then complementary DNA (cDNA) was synthesized using the FastQuant RT kit (Tiangen Biotech Co., Ltd, China). The synthesized cDNA was subsequently amplified with specific primers and SuperReal PreMix Plus (Tiangen Biotech Co., Ltd, China) using the IQ5 Multicolor Real-Time PCR Detection System (BioRad Laboratories, Hercules, Calif). Primers with the following sequences were purchased from Sangon Biotech Co., Ltd. (Shanghai, China): SIRT3, GCCTCTACAGCAACCTTCAGCAG and GAAGCAGCCGAAGGAAGTAGTGAG; PGC‐1α, CGGTGGATGAAGACGGATTGCC and ATTGTAGCTGAGCTGAGTGTTGGC; NRF1, TCTGCTGTGGCTGATGGAGAGG and GATGCTTGCGTCGTCTGGATGG; TFAM, CCGGCAGAAACGCCTAAAGA and ATCCTTAGCCCCCTGGAAGC; and GAPDH, GCAAGAGAGAGGCCCTCAG and TGTGAGGGAGATGCTCAGTG. Relative changes in gene expression were calculated according to the 2−ΔΔCT method, and all experiments were repeated at least three times (n = 3).

### Protein Extraction and Western Blotting

Myocardial tissues were cut into pieces and homogenized in lysis buffer containing 100× phosphatase inhibitor, 100 mM PMSF, and 1,000× protease inhibitor (KeyGen Biotech, Inc, Nanjing, China). Subsequently, the homogenization solution was transferred to a new tube and centrifuged at 12,000 g for 5 min at 4°C. The concentrations of the supernatants were measured using a BCA protein assay kit (Beijing Solarbio Science & Technology Co., Ltd, China). Mitochondrial protein was extracted according to the tissue mitochondrial isolation kit protocol (Beyotime Biotechnology, Shanghai, China). Briefly, tissues were shredded, digested with trypsin, homogenized on ice, and centrifuged at 600 g for 5 min at 4°C to collect supernatants containing the mitochondria. Subsequently, the lysates were centrifuged at 11,000 for 10 min at 4°C, and resulting supernatants containing the mitochondrial protein lysate were recovered. Cytoplasmic protein was extracted according to the standard protocol from KeyGen Biotech, Inc (Nanjing, China). In brief, tissues were homogenized, placed on ice for 30 min, and centrifuged at 3,000 g for 10 min at 4°C. The supernatant contained cytoplasmic protein.

To perform immunoblotting, proteins were separated by SDS-polyacrylamide gel electrophoresis (PAGE), followed by transfer onto polyvinylidene difluoride (PVDF) membranes. Then, the membranes were blocked with 5% defatted milk for 2 h at room temperature and incubated overnight at 4°C with primary antibodies diluted in TBST. The following primary antibodies were used: SIRT3 (1:1,000, Proteintech Group, Inc, # 10099-1-AP), PGC-1α (1:1,000, Proteintech Group, Inc, #66369-1-Ig), NRF1 (1:500, BOSTER, #AO1129-2), TFAM (1:500, BOSTER, #PB0413), Pro-Caspase-3 (1:1,000, bioss, # Bs-2593R), Cleaved Caspase-3 (1:1,000, Affinity, # AF7022), Cyt C (1:800, Proteintech Group, Inc, # 10993-1-AP), COXIV (1:1,000, Proteintech Group, Inc, # 11242-1-AP), and GAPDH (1:1,000, Cell Signaling Technology, #5174). After washing with TBST 3 times, the membranes were incubated with peroxidase (HRP)-conjugated secondary antibody and visualized using luminol reagent (Engreen Biosystem Co, Ltd., Beijing, China) (n = 3).

### Statistical Analysis

All experimental data are expressed as means ± S.D. SPSS 19.0 (Lead Technologies, Chicago, Illinois) was utilized for statistical analyses. Significance was determined by one-way analysis of variance (ANOVA), followed by Tukey’s multiple comparison test. A difference of P < 0.05 was considered statistically significant.

## Results

### PER Mitigates Cardiac Dysfunction in Rats Induced by ISO

**Figure 1:**
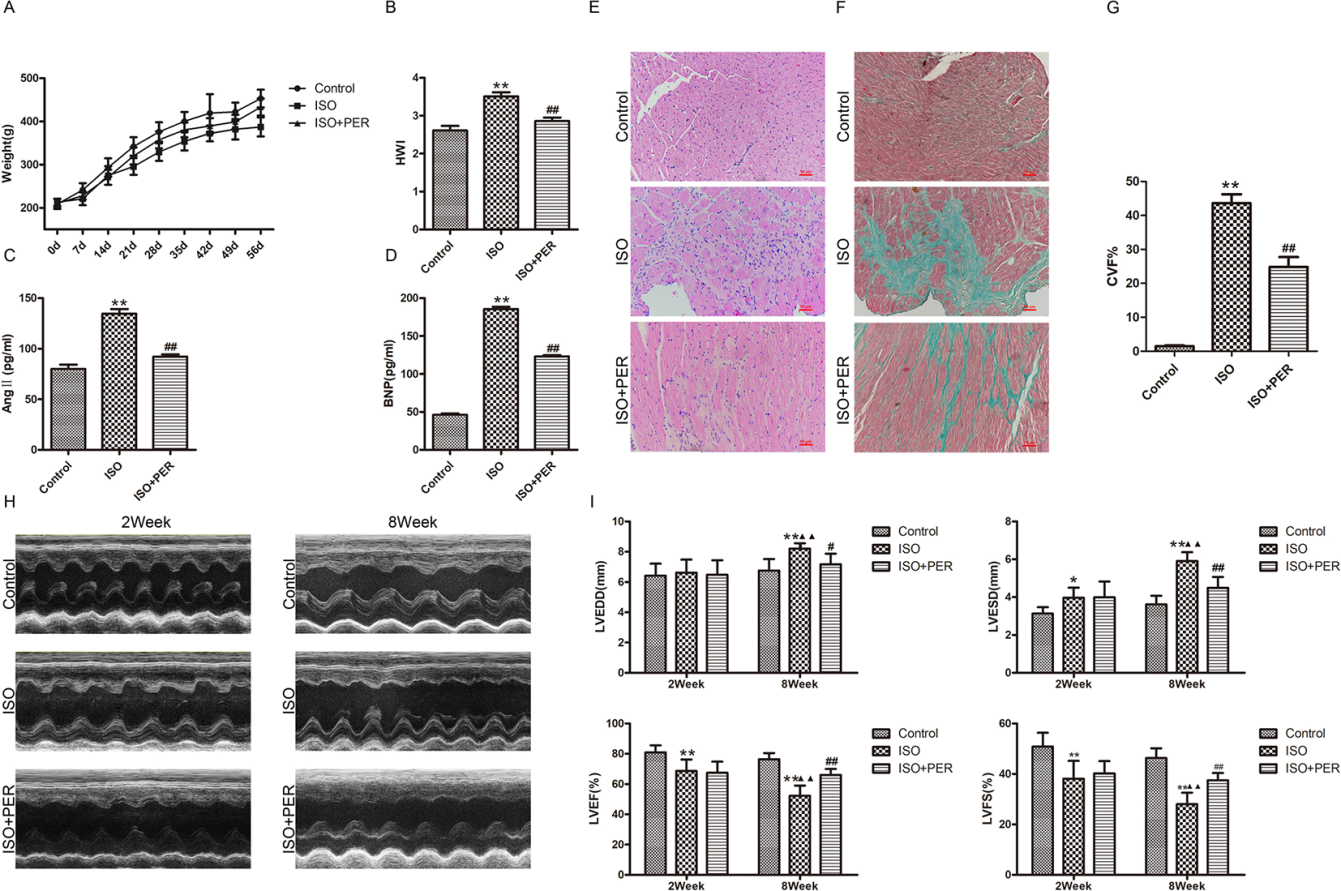
Perindopril (PER) enhances heart function in isoproterenol (ISO)-induced cardiomyopathy model rats. **(A)** Changes in body weights for control rats and ISO-induced cardiomyopathy rats treated with out without PER (n = 10). **(B)** Changes in rat heart weight index (HWI) for different groups (n = 10). **(C)** Ang II levels in sera were measured by ELISA assay (n = 6). **(D)** B-type natriuretic peptide (BNP) levels in sera were measured by ELISA assay (n = 6). **(E)** Histopathological changes in ventricular myocardial tissues observed by H&E staining (×200 magnification) (n = 5). **(F)** Masson’s Trichrome Staining of cardiac tissues (×200 magnification). Blue color represents fibrosis (n = 5). **(G)** The Collagen Volume Fraction (CVF) of myocardial tissues was calculated as quantification (n = 5). **(H)** Representative M-mode echocardiograms for ISO, ISO + PER and control groups (n = 6). **(I)** Bar charts showing LVEDD, LVESD, LVEF%, and LVFS%. Data are represented as mean ± SD, #P < 0.05, ##P < 0.01 compared with ISO group, *P < 0.05, **P < 0.01 compared with control group, ▲▲P < 0.01 compared with Week 2, LVEDD: Left ventricular end diastolic diameter; LVESD: Left ventricular end systolic diameter; LVEF%: Left ventricular ejection fraction; LVFS%: Left ventricular fractional shortening (n = 6).

To assess the mechanism of PER in improving cardiac health, we established an ISO-induced rat cardiomyopathy model. According to **Figure 1A**, ISO stimulation markedly slowed down weight gain, while PER treatment reduced the effect of ISO. In addition, the hearts in the ISO group were remarkably expanded as assessed by the HWI, whereas the expansion was less dramatic after PER treatment (P < 0.01) (**Figure 1B**). Evaluation of the serum biochemical index levels suggests that ISO induced BNP and AngII; however, PER intervention restrained the secretion of these proteins, which suggests that PER mitigates the activation of the neuroendocrine system (**Figures 1C, D**). Furthermore, H&E staining results demonstrate that ISO induced abundant inflammatory cell infiltration, disordered cardiac muscular fibers, and cardiac hypertrophy, and these pathological morphological effects were reduced in the ISO + PER treatment group (**Figure 1E**). Masson's trichrome staining further revealed that the ISO group had disorderly arranged myocardial tissues, along with large areas of congestion and irregular collagen fibers. However, the amount of fibrotic connective tissues was dramatically reduced in the ISO + PER group (**Figures 1F, G**), which is consistent with the H&E staining results.

Variations in heart function, shape, and size were further evaluated by echocardiography. In comparison with control group, animals treated with ISO displayed ventricular remodeling, which was apparent after week 2 and further enhanced at the end of week 8 (**Figures 1H, I**). This was accompanied by a decline in cardiac function, as evidenced by increased LVESD and LVEDD levels and decreased LVEF and LVFS levels after week 8 (**Figures 1H, I**); however, PER attenuated these pathological changes. These results verify that PER attenuates the effect of ISO in inducing cardiomyopathy.

### PER Mitigates OS and Enhances Mitochondrial Function in Myocardial Tissues of ISO-Induced Rats

**Figure 2:**
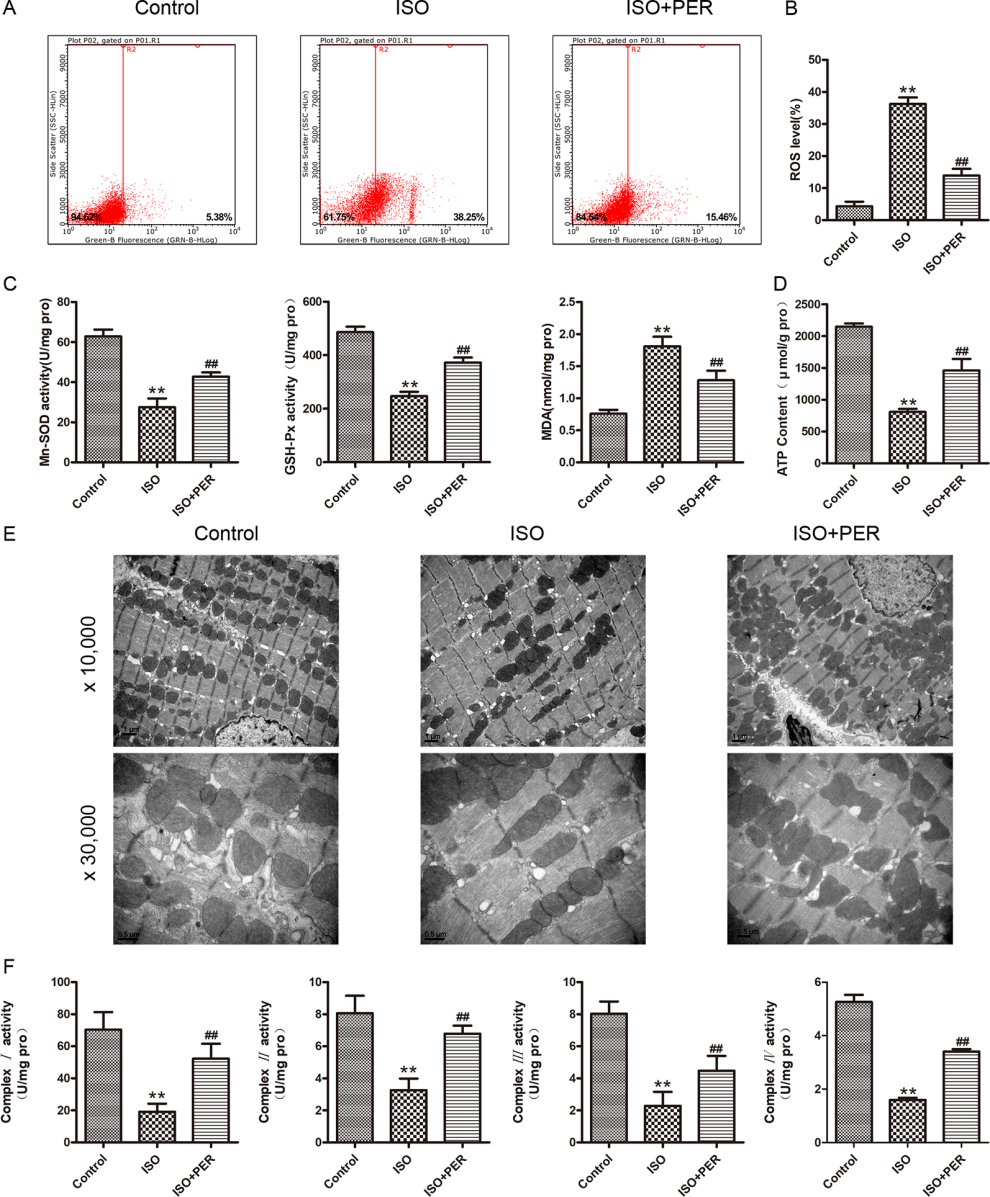
Perindopril (PER) attenuates myocardial mitochondrial function and oxidative stress in isoproterenol (ISO)-induced cardiomyopathy rats. **(A)** Flow cytometry was performed to analyze the total reactive oxygen species (ROS) levels in podocytes based on DCHF-DA staining (n = 3). **(B)** Quantitative analysis of ROS (n = 3). **(C)** Myocardial tissues were homogenized, and then GSH-px, Mn-SOD, and malondialdehyde (MDA) levels were assessed (n = 6). **(D)** ATP content in myocardial tissues was assessed (n = 6). **(E)** Cardiac ultrastructure of myocardium examined by electron microscope, magnification 10,000× and 30,000× (n=3). **(F)** Mitochondria were isolated from myocardial tissues for detection of complexes I, II, III, IV activities (n = 6). Data are represented as means ± SD, ^##^P < 0.01 compared with the ISO group, and **P < 0.01 compared with the control group.

To determine whether the mechanism of PER in attenuating cardiomyopathy involves changes in the OS level, we examined the antioxidant enzyme activity and ROS contents in the myocardial tissues of rats. ISO stimulation markedly increased myocardial OS, as verified by elevated ROS levels; and PER intervention reduced this increase (P < 0.01; **Figures 2A, B**). Consistently, ISO reduced the activities of the mitochondrial antioxidants MnSOD and GSH-Px and increased the levels of the OS marker MDA; and PER partially reversed these changes (P < 0.01; **Figure 2C**). These results suggest that PER may function by attenuating the mitochondrial stress caused by ISO.

To further assess this possibility, we evaluated the effects of ISO and ISO + PER on mitochondrial ATP levels. ISO significantly decreased the mitochondrial ATP content, and PER attenuated this decrease (P < 0.01; **Figure 2D**). To demonstrate the effect of PER on ATP production, an electron microscope was used to examine the ultrastructure of the mitochondria. Swollen and fragmented mitochondria were observed to be evident in ISO group, and the cristae in the mitochondria appeared distorted and in some cases were completely lysed. However, the disruption of mitochondria was markedly ameliorated after PER treatment. Moreover, mitochondrial number was less in the ISO group compared to the control group, and was greater in the PER treatment group (**Figure 2E**). To verify that these effects on mitochondrial ATP content were mediated at the level of ATP synthesis, we evaluated the activities of the mitochondrial respiratory chain complexes I, II, III, and IV. The activities of each of these complexes were significantly reduced in comparison with those of the control group; however, PER statistically reversed the decreases (P < 0.01) (**Figure 2F**). These results demonstrated that PER intervention protects the dysfunction of the electron transport chain in rat myocardial cells caused by ISO induction, thus decreasing ROS production and elevating ATP synthesis.

### PER Decreases ROS-Derived Myocardial Apoptosis in ISO-Induced Rats

**Figure 3:**
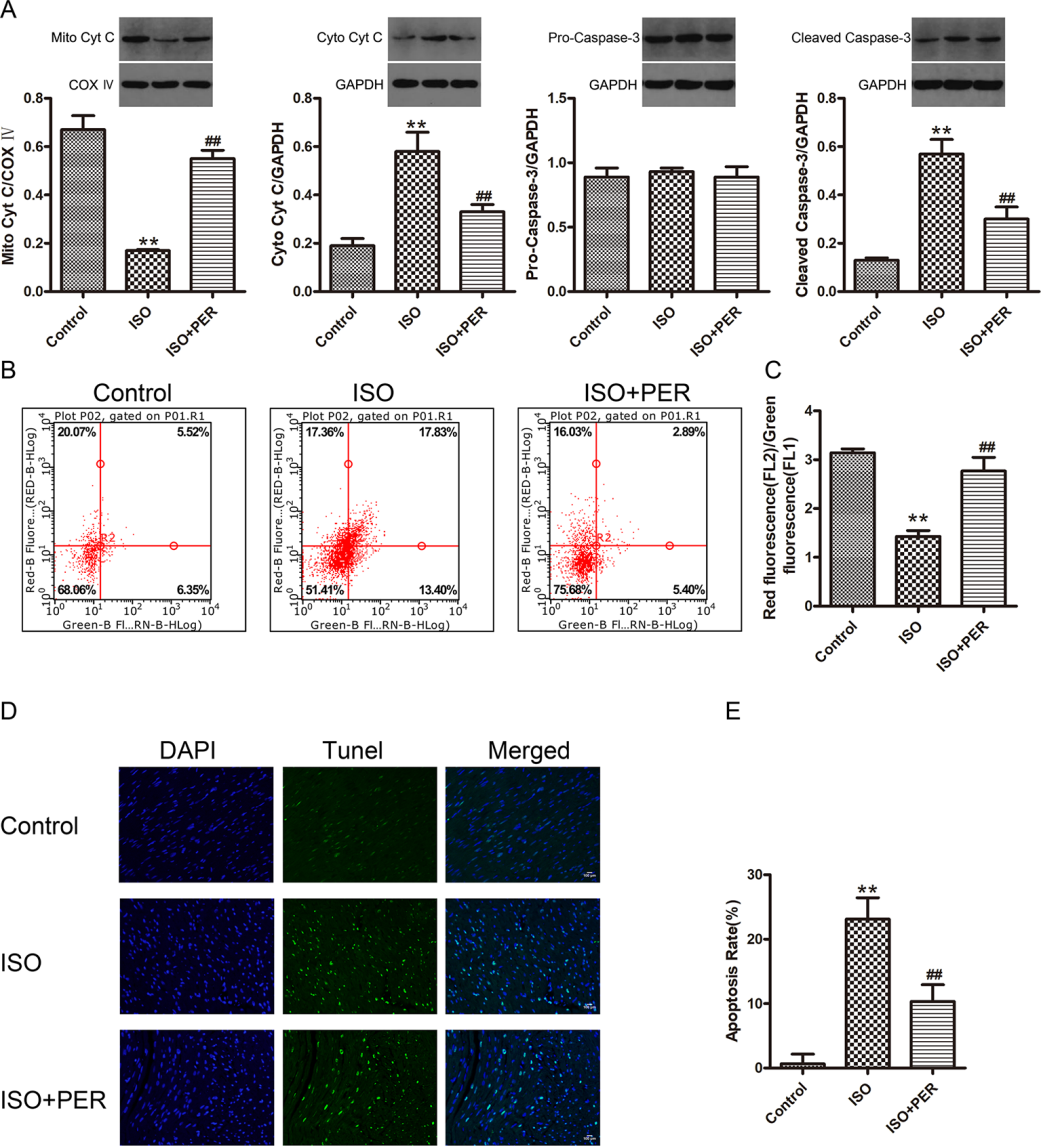
Perindopril (PER) attenuates myocardial apoptosis in isoproterenol (ISO)-induced cardiomyopathy rats. **(A)** Western blotting of Cyto Cyt C in cytoplasmic lysates, Mito CytC in mitochondrial lysates, and Cleaved Caspase-3 and Pro-Caspase-3 in total cell lysates (n = 3). **(B)** Flow cytometry was employed to analyze the mitochondrial membrane potential (MMP) of myocardial cells based on JC-1 staining (n = 3). **(C)** The MMP was quantified as the red-to-green fluorescence ratio. **(D)** Representative DNA fragmentation assays measured by DAPI and TUNEL staining. **(E)** The apoptosis rate was computed as the positive cell percentage. Statistics were calculated from 5 views from every group (n = 5). Data are represented as means ± SD, ^##^P < 0.01 compared with the ISO group, **P < 0.01 compared with the control group.

To further assess the functional outcome of PER-mediated attenuation of OS, we evaluated the effects of PER on ISO-inducted myocardial apoptosis. ISO stimulation resulted in decreased mitochondrial cytochrome C (Mito Cyt C) levels, with a corresponding increase in cytoplasmic cytochrome C (Cyto Cyt C) levels, suggesting that the pro-apoptotic factor Cyt C leaked from the mitochondria to cytoplasm of myocardial cells in the ISO group. Furthermore, ISO treatment increased the levels of the apoptosis activator cleaved caspase-3, while PER suppressed both Cyto Cyt C leakage and caspase-3 activation (**Figure 3A**). To verify these findings, we assessed the effects of ISO and PER on the MMP. The myocardial MMP of the ISO group was notably lower than that of the control group (P < 0.01), while the MMP of the ISO + PER group was higher than that of ISO group (P < 0.01) (**Figures 3B, C**). Furthermore, TUNEL staining results confirmed that myocardial apoptosis was remarkably aggravated in the ISO group (P < 0.01), but that the apoptosis level was attenuated in the ISO + PER group compared to the ISO group (P < 0.01) (**Figures 3D, E**). These results suggest that PER reduces the level of myocardial apoptosis in the ISO-induced cardiomyopathy model.

### PER Modulates PGC-1α, SIRT3, TFAM, and NRF1 Protein and mRNA Levels in Myocardial Tissues From ISO-Induced Rats

**Figure 4:**
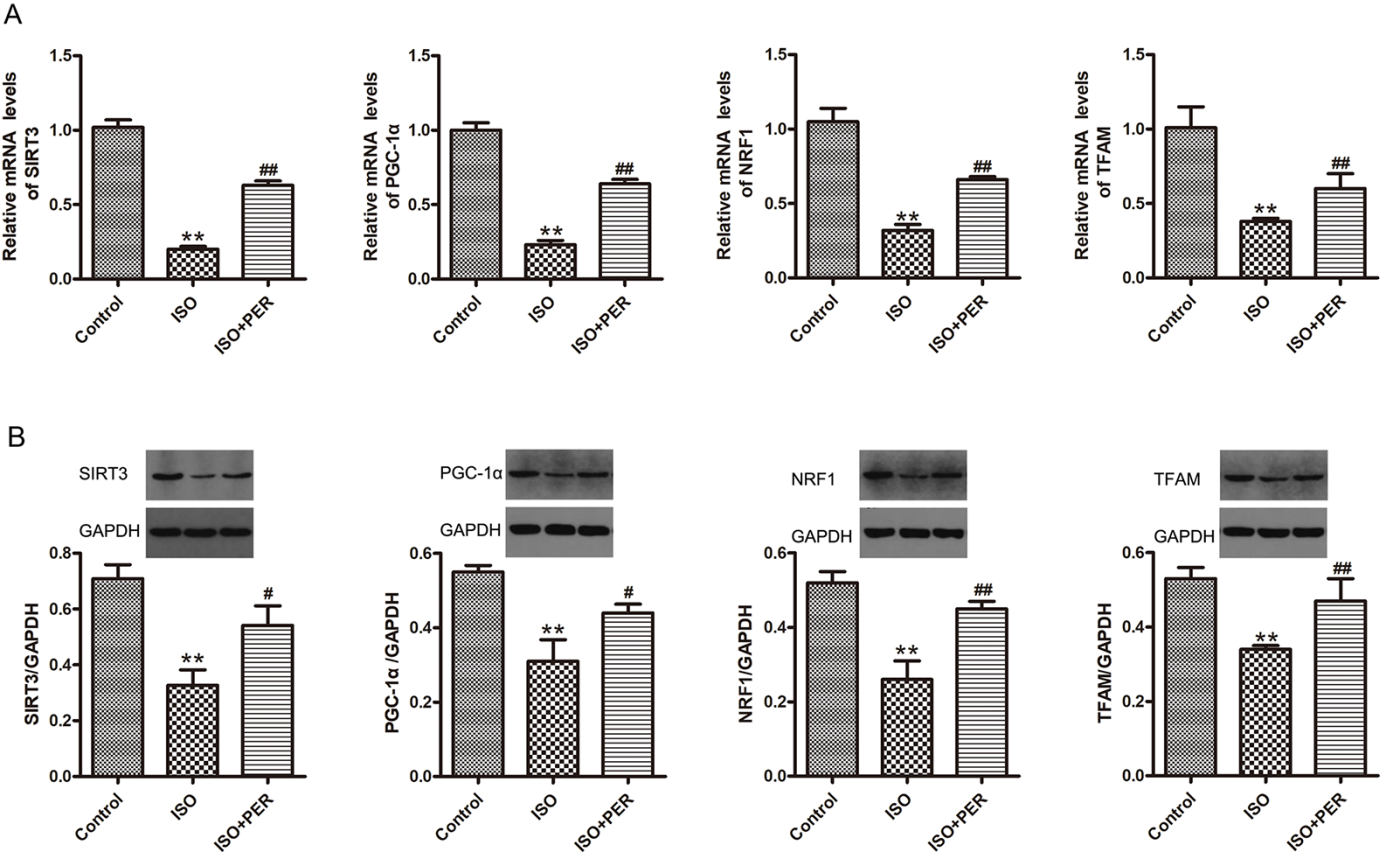
Perindopril (PER) restores the protein and mRNA levels of peroxisome proliferator-activated receptor gamma coactivator 1-alpha (PGC1α), Sirtuin 1 (SIRT1), mitochondrial transcription factor A (TFAM), and nuclear respiratory factor 1 (NRF1) in myocardial tissues from isoproterenol (ISO)-induced cardiomyopathy rats (n = 3). **(A)** Real-time PCR was carried out to analyze the mRNA levels of PGC1α, SIRT1, TFAM, and NRF1 within myocardial tissues (n = 3). **(B)** Western blotting of PGC1α, SIRT1, TFAM, and NRF1 within myocardial tissues (n = 3). Data are represented as means ± SD, ^#^P < 0.05, ^##^P < 0.01 compared with the ISO group, **P < 0.01 compared with the control group.

To determine whether PER might ameliorate cardiomyocyte dysfunction by improving mitochondrial biosynthesis, we performed Western blotting and RT-PCR of proteins/genes that are known to have a role in mitochondrial biosynthesis. ISO treatment markedly down-regulated the protein and mRNA levels of PGC-1α, SIRT3, mitochondrial transcription factor A (TFAM), and nuclear respiratory factor 1 (NRF1) (P < 0.01). Furthermore, the levels of each of these mitochondrial biosynthesis mediators were increased by PER intervention (P < 0.01) (**Figures 4A, B**). These findings suggest that PER enhances mitochondrial biosynthesis by activating the SIRT3/PGC-1α signal transduction pathway.

## Discussion

Despite the considerable efficacy of PER in improving cardiac function and increasing the survival after HF, little is known about its mechanism of action (Bertrand et al., 2009). In this study, we investigated the mechanism of PER in the ISO-induced myocardial damage rat model. Our experimental results demonstrate that PER attenuates myocardial remodeling in rats and prevents cardiac function decline, which might be related to its ability to up-regulate SIRT3 and PGC-1α. These findings provide greater insight into the functional responses to a proven effective pharmaceutical compound and suggest a direction for developing additional targeted therapies for clinical HF prevention and treatment.

Subcutaneous injection of ISO has previously been shown to induce the accumulation of catecholamine in animals, which thereby leads to cardiomyocyte hypertrophy, necrosis, and apoptosis, resulting in myocardial remodeling (Kazachenko et al., 2008). Therefore, this model involves a process of cardiomyopathy in animals that recapitulates well-established processes of human congestive HF (Parmley, 1992). In our study, the rat cardiomyopathy model was established by subcutaneous injection of ISO at 10.0 mg/kg/d for 2 weeks. According to the results of the HWI, serum biochemical indicators, echocardiography and histological analysis, ISO elevated serum AngII and BNP levels and stimulated myocardial necrosis, hypertrophy and fibrosis, accompanied by ventricular remodeling and a decline in cardiac function. However, the addition of PER reduced the serum AngII and BNP levels, arrested myocardial fibrosis, and inhibited the tendency of heart cavity enlargement. PER also improved cardiac function and delayed the occurrence of HF. Therefore, PER showed high efficacy in delaying and preventing features of HF in the ISO-induced rat model that are reflective of its therapeutic efficacy in congestive HF patients.

Recent literature (Lu et al., 2016; Wang et al., 2017; Cui et al., 2018) indicates that ISO stimulation increases the secretion of AngII, which is consistent with our experimental results. AngII has been shown to participate in OS by inducing ROS production (Privratsky et al., 2003). Therefore, we speculated that the cardioprotective effect of PER might be related to the inhibition of AngII-induced OS. Indeed, PER administration to ISO-induced rats markedly decreased the ROS content, increased the activities of Mn-SOD and GSH-Px, and lowered the *MDA content* in myocardial tissues. SODs are antioxidant enzymes that can eliminate superoxide anion free radicals and protect cells from oxidative damage (Chang et al., 2017), and Mn-SOD, which is located in mitochondria, mainly acts on the clearance of ROS in the mitochondria (Lu et al., 2019). Furthermore, GSH-Px has the function of scavenging lipid peroxides and forms an *in vivo* antioxidant defense system together with other oxidases (Lu et al., 2019). On the other hand, MDA is a product of lipid peroxide, and its levels are reflective of the degree of tissue oxidative damage. Therefore, each of these findings are consistent with the possibility that the cardioprotective effect of PER is related to the mitigation of OS, though the relative contributions of upstream regulators in this process remain to be fully elucidated.

The mitochondrial respiratory chain is known to be composed of five primary enzyme complexes for which structural damage or altered activity affects respiratory function: NADH-ubiquinone oxidoreductase, succinate-quinone oxidoreductase, ubiquinol-cytochrome C reductase, cytochrome c oxidase, and FOF1-ATP synthase. We usually refer to these enzymes as complex I, II, III, IV, and V. According to previous studies (Sun et al., 2016), the mitochondrial respiratory chain is one of the main sources of intracellular ROS. Under conditions of OS, elevated ROS production in mitochondria can result in dysfunction of the mitochondrial respiratory chain, leading to decreased ATP synthesis as well as activation of the endogenous apoptotic pathways (Yamagata et al., 2002; Munusamy and MacMillan-Crow, 2009). Dysfunction of the respiratory chain further increases ROS production, which creates a positive feedback loop and exacerbates the damage. Moreover, insufficient energy production and myocardial apoptosis are conducive to ventricular remodeling, ultimately leading to the occurrence of HF (Liu et al., 2017). The results of this study suggest that the increased ROS production in myocardial tissues of ISO-induced cardiomyopathy rats is accompanied by the destruction of the mitochondrial ultrastructure, decreased mitochondria numbers, reduced activities of mitochondrial respiratory chain I, II, III, IV, and decreased ATP production; however, PER intervention can ameliorate the disruption of mitochondria, attenuate decreases in mitochondrial numbers, improve respiratory chain function, and increase myocardial ATP content. Therefore, our findings support the identification of the mitochondria as a key site for PER-mediated cardioprotection.

Recent studies (Zhang et al., 2015; Liu et al., 2017) suggest that OS-induced production of excessive ROS induces apoptosis, and myocardial apoptosis causes ventricular remodeling, which ultimately leads to HF (Zhang et al., 2015; Liu et al., 2017). Furthermore, excessive production of ROS in mitochondria can cause damage to lipids and proteins of the mitochondrial inner membrane and induce intimal fluidity and permeability changes, including opening of permeability transition pores (mPTP) (Cao et al., 2011). This can rapidly destroy the MMP and change the osmotic pressure in mitochondria, causing membrane rupture, Mito Cyt C outflow, caspase-3 activation, and ultimately apoptosis (Cao et al., 2011). In this study, we demonstrated that PER treatment reduces cardiomyocyte apoptosis. Furthermore, the reduction of apoptosis is accompanied by increased MMP, inhibition of Cyt C release from the mitochondria into the cytosol and inhibition of caspase-3 activation. These results support our findings that PER reduces the activation of endogenous apoptotic pathways by reducing ROS production in mitochondria.

As the primary deacetylase in mitochondria, SIRT3 is a key regulator of cell energy metabolism, biosynthesis, and apoptosis. Previous studies (Torrens-Mas et al., 2019) have shown that SIRT3 regulates mitochondrial biosynthesis by activating PGC-1α. NAD+ is necessary as a cofactor for SIRT3 deacetylation (Klimova et al., 2019); as a result, SIRT3 activity can be regulated by NAD+, NADH and some NAD+ metabolites. In this study, SIRT3 expression was downregulated in the myocardial tissues of the ISO group, which could be related to an altered NAD/NADH ratio as a result of AngII stimulation. However, SIRT3 expression inhibition in damaged myocardium was restored by PER treatment. We observed a similar pattern of regulation for PGC-1α., NRF-1, and TRAM. PGC-1α is the most important mitochondrial biogenesis regulator and can regulate the expression of nuclear genome- and mitochondrial genome-related mitochondrial proteins through the PGC-1α-NRF-TFAM pathway (Cheng et al., 2016). Furthermore, both the mitochondrial respiratory chain enzymes and mitochondrial antioxidant factors (such as Mn-SOD and GSH-Px) are up-regulated by mitochondrial biogenesis (Chen et al., 2011; Sun et al., 2016). Therefore, these results indicate that PER improves mitochondrial biogenesis by up-regulating the expression of SIRT3 and the PGC-1α pathway.

As a limitation of our study, PER was used at 2 mg/kg. This is a standard dose that is effective in rat cardiomyopathy models (Weisinger et al., 2008; Tikellis et al., 2012; He et al., 2014; El-Shoura et al., 2019), but that may not be comparable to the dose used in humans. Furthermore, this study only used the ISO-induced cardiomyopathy model in rats. Future experiments with other cardiomyopathy models and varying doses of PER will be valuable for verifying the findings.

In conclusion, our results suggest that the myocardial remodeling and cardiac function decline in ISO-induced cardiomyopathy model rats may be associated with down-regulation of SIRT3 and PGC-1α expression. PER can improve mitochondrial biosynthesis and attenuate mitochondrial OS and apoptosis by restoring SIRT3 and PGC-1α expression, thereby improving cardiac function. These results provide increased understanding of the mechanism of PER in the prevention and treatment of HF in clinical practice.

## Data Availability Statement

All datasets generated for this study are included in the article/supplementary material.

## Ethics Statement

The animal study was reviewed and approved by The Animal Care and Use Committee of Tianjin Union Medical Center, Tianjin, China.

## Author Contributions

ZZ conceived and designed the study; collected, analyzed and interpreted the data; and assisted in writing the manuscript. HL conceived and designed the study and collected and presented the data. WC analyzed and interpreted the data. YC was involved in the animal experiments. AH assessed western blotting analyses. XQ conceived and designed the study, wrote the manuscript, and gave final approval of the manuscript.

## Funding

This research was supported by the Tianjin Municipal Bureau of Health for Science and Technology (Grant No. 2015KG110), the Tianjin Science and Technology Planning Project (Grant No. 16ZXMJSY00060), and the 2017 Annual Graduate Students Innovation Fund (Grant No. CXJJLX201710).

## Conflict of Interest

The authors declare that the research was conducted in the absence of any commercial or financial relationships that could be construed as a potential conflict of interest.

## Abbreviations

HF, heart failure; PER, perindopril; ISO, isoproterenol; ROS, reactive oxidative species; SIRT3, Sirtuin 3; PGC-1α, peroxisome proliferator-activated receptor gamma coactivator 1-alpha; NRF1, nuclear respiratory factor 1; TFAM, mitochondrial transcription factor A; NADH/NAD, nicotinamide adenine dinucleotide; AngII, Angiotensin II; OS, oxidative stress; SD, Sprague-Dawley; MMP, mitochondrial membrane potential; TUNEL, transferase dUTP nick end labeling; HWI, Heart weight index; BNP, B-type natriuretic peptide; MDA, malondialdehyde; GSH-Px, glutathione peroxidase; Mn-SOD, manganese superoxide dismutase.
